# Supplementary material for: Immune regeneration in irradiated mice is not impaired by the absence of DPP9 enzymatic activity
Source: Sci Rep. 2019 May 13;9:7292. doi: 10.1038/s41598-019-43739-w (PMC6513830; doi:10.1038/s41598-019-43739-w)
Supplement: Supplementary file 1 — Supplementary figures [file 41598_2019_43739_MOESM1_ESM.docx]

**Immune regeneration in irradiated mice is not impaired by the absence of DPP9 enzymatic activity**

Margaret G. Gall^*^, Hui Emma Zhang^*^, Quintin Lee, Christopher J. Jolly, Geoffrey W. McCaughan, Adam Cook, Ben Roediger and Mark D. Gorrell^#^

Affiliations: Centenary Institute, The University of Sydney Faculty of Medicine and Health, New South Wales, Australia.

**Supplementary Figures S1 to S4**

**Figure S1: Percentages of TER119^+^ and CD11b^+^ fetal liver cell populations without red blood cells exclusion, and flow cytometry gating strategies for fetal liver cells.**

**(A)** Fetal liver cells from DPP9^S729A^ and DPP9-WT (WT) embryos were stained with TER119-APC and CD11b-FITC antibodies and analyzed by flow cytometry without excluding red blood cells. No statistically significant difference was observed between the percentages of DPP9^S729A^ and WT cells in the inoculum for either erythroid lineage or myeloid lineage. Stacked bars and error bars represent mean and SD. Black circle and square represent individual data of TER119^+^ and CD11b^+^ cells, respectively. n = 5 (DPP9-WT embryos) or n = 5 (DPP9^S729A^ embryos). **(B)** Live (DAPI^–/lo^) cells were gated from time gate to remove potential fluidic issues and from two singlet gates, forward scatter (FSC) and side scatter (SSC), to remove doublets. DAPI^–/lo^ cells were then gated using FSC to exclude Red Blood Cells (RBC) and were subsequently gated to identify TER-119^+^ and CD11b^+^ cells. Plots showing live cells before RBC exclusion and FSC-A^lo^ cells after RBC exclusion are also provided. The representative gating is from a DPP9^S729A^ fetal liver sample.

**Figure S2: Body weight: primary chimeras.**

Irradiated mice inoculated with DPP9^S729A^ or DPP9-WT fetal liver cells were monitored for weight loss as a gross indicator of dysfunctional or failed immune regeneration. All irradiated mice exhibited an initial weight loss for several days before gradual weight increase back to the original start weight at about 4–5 weeks post-transplantation. Mean body weight of mice (**A**), and mean percentage weight change where 0 (red dotted line) represents the body weight immediately before irradiation (**B**). n = 7 mice per group.

**Figure S3: Body weight: secondary chimeras.**

Irradiated mice inoculated with DPP9^S729A^ (n = 10) or DPP9-WT (n = 9) bone marrow cells along with PTPRC^A^ WT bone marrow cells were monitored for weight loss as a gross indicator of dysfunctional or failed immune regeneration. All irradiated mice displayed an initial weight loss for several days before gradual weight increase, returning to the original weight by 2 weeks after irradiation. Mean body weight of mice (**A**), and mean percentage weight change where 0 (red dotted line) represents the body weight immediately before irradiation (**B**).

**Figure S4: Flow cytometry gating strategy for donor and recipient cell identification.**

Live (DAPI^–/lo^) white blood cells (WBCs) were initially gated from time gate to remove potential fluidic problems and two singlet gates to remove doublets. From live WBCs, CD45.1^+^ recipient cells and CD45.2^+^ donor cells identified, of which the donor cells were subjected to further gating. CD19^+^ B cells were selected, followed by CD19^–^NK1.1^+^ NK cells. From NK1.1^–^ population, CD3^+^ T cells and CD11b^+^ myeloid cells were gated. Finally, within the CD11b^+^ gate, Ly6G^+^ neutrophils were identified. This is a representative gating strategy from a GKI chimeric mouse 6 weeks post irradiation and transplantation.

**A**

**B**

Figure S1

**A**

**B**

Figure S2

**A**

**B**

**A**

**B**

Figure S3

Figure S4
